# Cincinnati Academy of Medicine

**Published:** 1888-06

**Authors:** E. S. McKee


### CINCINNATI ACADEMY OF MEDICINE.

Dr. E. G. Zinke, of Cincinnati, recently reported a case of Cæsarean section to the Cincinnati Academy of Medicine and discussed the question extensively, especially with reference to the Sænger operation.

REPORTED BY E. S. McKEE, M. D.

Dr. C. D. Palmer, in discussing the case, said that the paper possessed the qualities which both interest and instruct, and is one of more than ordinary value. Every such case should be reported, and when the report is as thorough and as painstaking as this one has been, it is a matter of congratulation for the Academy of Medicine.

Having been present at the post-mortem, he could testify to the accuracy of the pelvic measures as reported.

If classified, the pelvis might be called a justo-minor, except that the antero posterior diameter of the brim was contracted out of all proportion to the remaining diameters. The contraction at the brim was about two inches at the conjugate, and 1 to 1¼ inches for the oblique diameters.

What was the proper management of the delivery in this and similar cases?

We have five different, distinct methods of delivery at our disposal in treatment of pelvic deformities, viz.: The induction of premature delivery, the forceps, version, craniotomy or its improved modification, and Cæsarean section or some of its modifications.

While in some cases these come in conflict, to a great extent, each has a special field of application. The question is, how do they apply to the particular case under consideration?

Although the question of the induction of premature labor has no direct bearing in this instance, at the same time it may be interesting to inquire whether this poor woman could not have been delivered of a living child, if the condition of her pelvis had been realized early enough in pregnancy, or whether, if she had recovered and again become pregnant, could she have been delivered *per vias naturales?*

Premature labor is induced for the sake of both mother and child from the 32d to the 36th week of utero-gestation; the earlier the better for the mother; the later the better for the child, *cæteris paribus.* Delivery has been induced as early as the end of the 7th lunar month, producing a living child, with a pelvis as small as 2.5 inches conjugata. But when we remember that the pelvis of the reported case was not only thus contracted in the conjugate, but also in all the other diameters, it implies that delivery would necessarily have to be induced prior to the 7th lunar month. It would probably have to be completed by the forceps, or version, with some delay and at a time when the child was very young and feeble. It seems barely possible that it could have been born alive, or if alive for it to survive on account of prematurity. Therefore it is safe to say that the induction of premature labor was in this case impossible with the hope of a living child.

If this woman had survived and again become pregnant, the choice between two modes of procedure would have had to be made—the induction of abortion early in the pregnancy, or better than that, the allowing of the pregnancy to go on to term and then, at a favorable time in labor, to perform abdominal and uterine section.

This was no case for the forceps; the range of this instrument is for pelves from 4 inches to 3.5 or 3¼ in conjugate. He would not wish to condemn the practice of the gentleman who used forceps in this case, hoping thereby to effect delivery. Under the existing circumstances, almost any obstetrician would have applied the forceps at first to see what they could do. Lack of knowledge, with the exact pelvic dimensions to be encountered, could only be supplied by the experience then and there obtained.

Nor was this a case for the performance of podalic version. The range of application of version is in pelves with conjugate diameters capable of delivering a living child, and in proper cases is a most useful method of improvement upon nature's mechanism, as well as the utilization of additional forces to aid the expulsion of the child.

The conjugate diameter of the pelvic brim is more frequently and decidedly contracted in pelvic deformities than any other; therefore its measurements have served as the chief guide, the key to the lock of the pelvis. A conjugate ranging from 2.75 inches to 1.75 inches is the field for the utilization of craniotomy and Cæsarean section. The conjugata of the pelvis under discussion was 2.5 inches, and within this range. Manifestly, then, one or the other of these two methods was to be considered. A live child at term could not have been delivered *per vias naturales*, and if the child were to be saved Cæsarean section offered the only resource.

As a general rule, we may say, if the child is dead, with these measurements, craniotomy is the proper resort; if alive, Cæsarean section; but no absolute, inflexible rule can be laid down; there are always exceptions. Under certain conditions, craniotomy may be the best, though the child is alive, and Cæsarean section is to be preferred with a dead child if the pelvic contraction is very great.

To a great extent, Cæsarean section is the operation of selection, while craniotomy is the operation of necessity.

The interests of the mother are not tantamount but paramount. In all cases we are first to look to the mother; though this principle in management is old, it stands as strong to-day as it ever did.

While a mother is undoubtedly called upon to sacrifice somewhat of her chances for life in order that she may have a living child, she should not be asked to sacrifice life itself. No woman in parturition should be advised to submit to operative procedures which place her life in great jeopardy for the sake of her child if there be a more favorable way. Unless there be a reasonable prospect of saving her life as well as her child's, the Cæsarean section should be ruled out. She has the right to decide.

In a recent discussion of this subject, Dr. Meadows, one of the most eminent of the obstetricians of Great Britain, exclaimed: "Where now is craniotomy?" In reply, the speaker would remark, it still lives, and should live.

The question of the risks of each in determining between those two operations should be next then to the life of the child.

With pelvic contractions, not greater than 2.5 inches, the risks of craniotomy are not great, less than by Cæsarean section; if done early very few need die. Death after craniotomy is largely due to delays and other manifestations. But the risks rapidly accumulate; when the contraction extends below 2.5 inches, they may exceed those attending Cæsarean section. All things being equal, as to the time of operations and conditions, it may be said that the risks of the two operations are about the same with a pelvic measurement at 2.5 inches; at this point the two operations must come into severe competition. As the mother's interests are paramount, craniotomy is not to be laid aside unless

Cæsarean section holds forth equally good chances for her life.

Although the child is living, the following is a condition in which craniotomy is justifiable:

A long, difficult labor extending over many hours, perhaps days. All the liquor amnii has drained off; the uterine discharges are dark, unhealthy-looking, offensive. The fœtal head is impacted within the pelvis or it may be floating over the brim; cervix is swollen, œdematous, bruised; vagina swollen, livid. The uterus is tetanic or has ceased to act. The patient exhausted, anxious face, frequent pulse, feverish. This is no uncommon picture. What conditions are these for uterine section? What chance has such a woman for recovery after Cæsarean section? In what condition is such an uterus to be cut, to contract, to be sewed, to heal? Craniotomy offers a far better chance for the mother unless the contraction is at or below two inches.

Much of the opposition to craniotomy has arisen from two reasons: First, because it has been performed too frequently, and of course unjustifiably; but abuse is no argument for its total abolition. Second, because of the very favorable reports attending Cæsarean section, especially in Germany. In the hands of Sænger, Martin, Leopold, Crede and a very few others, the recovery has been nearly 80 per cent. If we contrast this high rate of recovery in Germany with the rate in the United States, it speaks very unfavorably for us. Out of nearly 160 cases collected by Robert Harris, the per cent. of recoveries is only 87.5. These figures must not simply be taken on their face, however, without inquiry into the attending causes and circumstances.

In the first place, the operations in Germany are done by a few experienced and skillful men. Most of them have been performed in hospitals with good surroundings. They have been done early in labor at the most favorable time. As a rule the exact opposite is true in the United States. No better proof is offered of the earliness of the operations done abroad than out of 88 cases 81 children were born alive. If we select the cases in which the operations were performed early in the United

States, we will see that the rate of recovery is very good, viz., about 70 per cent.

Great stress has been placed by Sænger upon his method of suturing. While the speaker would not wish to detract one iota of merit justly due this faithful and deserving worker, yet nevertheless it must be apparent to the careful investigator that his great success is not attributable so much to this as the early performance of his operations. The Sænger operations in this country (5 in number) have all proven fatal because they were done late. The Sænger *technique* could not save them—they died despite the operation.

The three great factors in the success of Cæsarean section are first, that it is to be done early while the general condition of the patient is still good, and while the local conditions are favorable; second, it must be done antiseptically; third, the uterine wound must be most carefully sutured. The first factor is the most important of all.

So long as the dangers of Cæsarean section exceed those of craniotomy, so long will the conflict between them continue. When the *technique* of the Sænger operation is completed, when this *technique* is well understood, when the operation is performed early, when pelvic deformities are more thoroughly studied and appreciated in this country, then will the section be more frequently made, its rates of success gradually increase, and in the same ratio will the necessities for craniotomy diminish. We will then be obliged to encounter fewer of those dreadful cases when, at our first interview, we are brought face to face with a parturient woman, exhausted after several days' labor, a midwife and several practitioners in attendance, each wearied out with fruitless efforts to deliver, soft parts bruised, swollen and lacerated, and finally a craniotomy or section with a forlorn hope.

The abolition of craniotomy on living children is a consummation devoutly to be wished, but, in the present state of affairs, is an advance not yet to be realized.

In the case of Dr. Zinke, conditions were certainly unfavorable for Cæsarean section, still it was probably to be preferred to craniotomy because the child was living, and the pelvic measurements were at that border-line where the risks of the two operations did not widely differ. Of course it will now remain an open question whether this woman might not have recovered under craniotomy.

The speaker did not regard this as a proper case for the Porro operation. The uterus was in fair condition for section, and this is always to be preferred, except in those instances where the organ is bruised, lacerated, gangrenous or septic. These complications may make the Porro-Muller operation not only superior but necessary.

Laparo-elytrotomy possesses certain advantages in the diminished risks of a loss of blood and septic infection, but its *technique* is not so well understood or so easy of execution. The improved section is generally preferable.

The improved Cæsarean section is the outgrowth of many advances in abdominal surgery running through a long period of years. The Sænger method has nothing distinctly original due to Sænger, but embraces points introduced and executed by Muller, Frank, Leopold, Kehrer and others. Sænger has, nevertheless, formulated all these improvements and shown their combined utility; hence, with perfect propriety, the operation, as now understood and now recognized as the best, ought to be called the Sænger.

In conclusion the speaker said that the Cæsarean section had only, to his knowledge, been performed three times in Cincinnati—once by Dr. Walton on a dwarf, done early in labor, once by Dr. Dandridge (laparo-elytrotomy) done late, and, finally, the case reported this evening by Dr. Zinke. All were fatal.

